# Surgical Aspect of Facial Nerve Disorders

**DOI:** 10.1155/2012/237631

**Published:** 2012-03-25

**Authors:** Sertac Yetiser, Peter S. Roland, Nebil Goksu

**Affiliations:** ^1^Department of Otorhinolaryngology and Head-Neck Surgery, Anadolu Medical Center, 41400 Kocaeli, Turkey; ^2^Department of Otorhinolaryngology and Head-Neck Surgery, University of Texas Southwestern Medical Center, Dallas, TX 75390, USA; ^3^Department of Otorhinolaryngology and Head-Neck Surgery, Medical School, Gazi University, 06500 Ankara, Turkey

Facial expression is the essential complementary of the verbal communication between humans. “Face is a window to the heart.” An ancient proverb tells us the importance of facial expression more than anything. When harmonic and symmetric movement of both sides of the face has lost, one is unable to express his emotion by distorted facial movement. This person becomes unwilling to communicate, hides himself, and escapes from social events. It is a collapsing life for patients not being able to cope with physical and psychological consequences of a facial paralysis.

Facial nerve is one of the unique cranial nerves innervating several tiny muscles and maybe the original one traveling through a long bony tunnel. These particular features make this nerve more vulnerable to different injuries than others resulting in obvious involvement of several agonist and antagonist muscles. An injury which may not be so harmful for others may cause a long-term bothersome problem by entrapment of axonal conduction. Thereafter critical questions directed by the patients are as follows: Is the facial function expected to recover soon, to what extent, and when? The primary problem facing the clinician is to distinguish the patients who will recover spontaneously or with medication from those who will not.

Facial nerve dysfunction can be seen in a sudden or gradual manner. However, investigation of possible underlying causes as well as a prognostic evaluation is necessary. For more chronic problems, a multidisciplinary team work provides better solutions for the relief of symptoms. Protection of eye, prevention or treatment of synkinesis, and resolution of psychological problems are best handled with collaboration between specific experts. As a summary, acute facial nerve dysfunction, chronic facial nerve problems, and its several presentations can be managed by medical and surgical ways. However, there will always be a new approach to this old problem.

Restoration of functional integrity of the diseased facial nerve has been subject of studies for decades. This special issue about facial nerve problems, probably, will not be the last one. However, prognosis of facial nerve injury, selection of good candidates for surgery, decision making and timing, as well as the type of approach always need new updates and renewals. This special issue will give some insight to this problem and promote a discussion of some different aspects of the facial paralysis.

*Sertac Yetiser* *Sertac Yetiser* *Peter S. Roland* *Peter S. Roland* *Nebil Goksu* *Nebil Goksu*

